# A Contemporary Review of Clinical Factors Involved in Speech-Perspectives from a Prosthodontist Point of View

**DOI:** 10.3390/medicina59071322

**Published:** 2023-07-18

**Authors:** Dana Gabriela Budală, Costin Iulian Lupu, Roxana Ionela Vasluianu, Nicoleta Ioanid, Oana Maria Butnaru, Elena-Raluca Baciu

**Affiliations:** 1 Department of Implantology, Removable Prostheses, Dental Prostheses Technology, “Grigore T. Popa” University of Medicine and Pharmacy, 16 Universității Street, 700115 Iași, Romania; dana-gabriela.bosinceanu@umfiasi.ro (D.G.B.); iulian.lupu@umfiasi.ro (C.I.L.); elena.baciu@umfiasi.ro (E.-R.B.); 2 Department of Fixed Prosthodontics, Faculty of Dental Medicine, “Grigore T. Popa” University of Medicine and Pharmacy, 16 Universității Street, 700115 Iasi, Romania; 3 Department of Biophysics, Faculty of Dental Medicine, “Grigore T. Popa” University of Medicine and Pharmacy, 700115 Iasi, Romania; oana.maria.butnaru@umfiasi.ro

**Keywords:** complete denture, phonation, speech

## Abstract

*Background and Objectives:* Learning to speak properly requires a fully formed brain, good eyesight, and a functioning auditory system. Defective phonation is the outcome of a failure in the development of any of the systems or components involved in speech production. Dentures with strong phonetic skills can be fabricated with the help of a dentist who has a firm grasp of speech production and phonetic characteristics. Every dentist strives to perfect their craft by perfecting the balance between the technical, cosmetic, and acoustic aspects of dentistry, or “phonetics”. The ideal prosthesis for a patient is one that not only sounds good but also functions well mechanically and aesthetically. Words are spoken by using articulators that alter their size and form. *Conclusions*: Therefore, a prosthesis should be made in such a way that it does not interfere with the ability to communicate. As a result, a prosthodontist has to have a solid grasp of how speech is made and the numerous parts that go into it.

## 1. Introduction

The ability to express oneself verbally is fundamental to human social interaction, and identity construction and is fundamental to social life. A denture’s effectiveness in conversation depends on more than just its functional and cosmetic qualities [1,2]. The neuromuscular system, acoustic phenomena, and the exact synchronization of oral structures are all required for its generation [2,3].

Speech is often regarded as one of the most difficult verbal tasks. It is an oral motor activity similar to mastication in many ways [4]. The vibration of the vocal cords is what makes the sound when air travels from the lungs to the trachea to the larynx. Both the static (the teeth and hard palate) and dynamic (the lips and tongue) articulators amplify and alter the sound further [3,4]. That is why articulator location is such a defining feature of speech sounds [1,3,4]. Changes therein may degrade the quality of speech articulation, leading to mistakes in the production of certain speech sounds [3,5,6].

Neural, muscular, mechanical, aerodynamic, acoustic, and auditory components are all necessary for speech production [1]. A prosthodontist’s familiarity with phonation principles is crucial for the successful delivery of a denture that restores or facilitates natural speech. Due to the mouth’s central role in phonation, a denture will have obvious effects on a person’s ability to communicate [7].

Prosthodontists have a constant challenge due to the intricacy of the mouth cavity and surrounding regions. Actions that are essential for survival, such as breathing, swallowing, chewing, facial expression, and communication, are examples of functional motions. Thus, prosthodontists have a moral obligation to restore their patients’ ability to communicate by restoring their lost teeth, but they must also take a comprehensive approach to ensure that their patients are able to lead trouble-free lives after receiving prosthetic restorations.

This study will concentrate on only one of these functions—speech—in order to understand the anatomical and physiological bases for how speech is connected to prosthodontics.

## 2. Literature Review

### 2.1. Mechanism of Speech

The study of the acoustics of the human voice is known as phonetics. There are three systems required for speech production [3].

**Figure 1:**
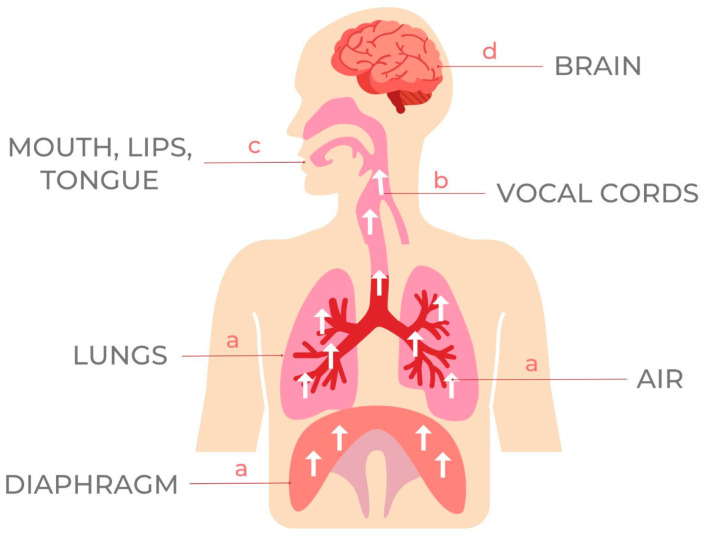
Mechanisms involved in speech activity: (a) diaphragm lungs and air stream-creating energy; (b) source of sound-vocal cords; (c) vocal apparatus-mouth, lips, tongue; (d) central coordinator-brain.

The first is a source of energy. For human speech sounds, the air flowing from our lungs provides energy. The second is the source of the sound: air flowing from the lungs arrives at the larynx. Third, the motor system uses the vocal apparatus—the lips, tongue, palate, and teeth—to articulate the generated sounds into words by obstructing, constricting, or diverting the airstream in a specific way [3,4], as represented in Figure 1.

The initiator is the brain region responsible for motor speech, along with the neural networks that carry these impulses to the organs of speech. The initiator plays a crucial role in the neurotic or nervously ill patient who appears to be having trouble even when the denture is phonetically correct [8,9].

### 2.2. Factors Influencing the Speech

Communicating verbally is fundamental to being human. The tongue has several interactions with the teeth, alveolar ridge, and hard and soft palates during speech sound creation. Proprioceptive feedback may be altered when these components are either covered or replaced by a denture. Therefore, dentures may alter the phonetics [10].

Since a denture’s success depends on a number of elements, including mechanics and aesthetics, phonetics must also be taken into account [11,12,13]. Poorly fitting and poorly retained removable complete dentures (CDs) can alter articulation points on the palate and anterior teeth, reduce tongue mobility, and narrow the oral cavity, all of which can contribute to speech articulation disorders [14,15]. Understanding the basic mechanism of speech and the specialized mechanics of the production of sound units of dental concern is necessary for handling the phonetics problem.

#### 2.2.1. Tongue

Phonetics primarily focuses on how the flow of air alters as it travels through the mouth and throat. Therefore, we are especially interested in the enunciators. The tongue is especially important among them. For each vowel sound, the tongue assumes a little different position and shape than it does while pronouncing a consonant. The tongue makes contact with the teeth, alveolar ridge, or hard palate in order to form each consonant during speech. Dentures can either cover or replace these structures; therefore, it is important for the dentist to know where the tongue contacts them [11,12].

When it comes to teeth, it is the consonant sounds that really matter. According to the anatomical structures involved in their development, they can be categorized [13] as follows, in four main types of speech sounds (Table 1).

##### Palatolingual, Made with the Tongue and Hard or Soft Palate

**Figure 2:**
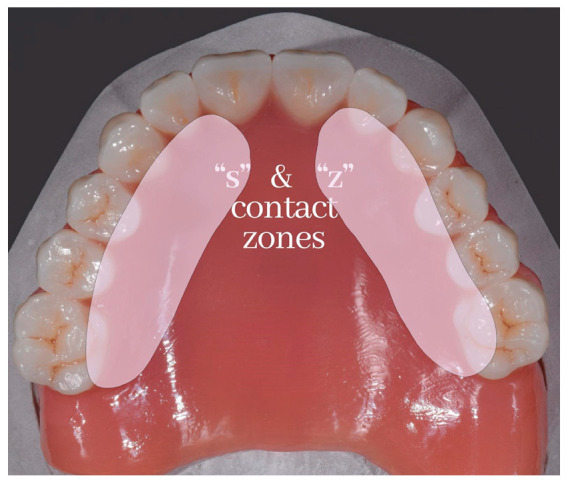
Areas involved in sound “s” and “z” production.

When the tongue is placed directly below the upper incisor teeth, the hiss of air escaping from the median groove of the tongue produces the sound “s”, as in sixty-six. Upper posterior teeth and alveolar ridges are in touch with the sides of the tongue, and this contact may go forward to the area of the lateral incisors (Figure 2) [16].

The right grooving of the tongue is crucial in the production of a “s” sound. As the depth of this groove is reduced, the sound of “s” is mellowed towards “sh”, and as the depth is reduced even further, the sound of “s” is mellowed towards “th” (as in a whisper). Lisping is commonly brought on by an overly thick denture base at the front of the palate. Whistling may occur if the patient has a tongue grove that is excessively deep. Whistling and lisping are polar opposites. Denture bases can be thickened in the right spot to reduce the depth of the groove of the tongue if the patient whistles.

The median raphe of the palate is not usually in the same place as the groove in the tongue. Therefore, the precise location of the groove in the tongue with respect to the palate should be identified if the patient has issues with the sound “s”. Finding this area of the palate allows the dentist to make adjustments to the denture base, such as making the groove deeper to address lisping or shallower to address whistling. Using the upper trial denture, a palatogram may be created to pinpoint the exact location of the tongue groove with respect to the palate.

The “s” sound also depends on the placement of the frontal teeth. Since the tongue is packed posteriorly due to the position of the lower incisors, the sound of “s” is mellowed towards a lisp. On the other hand, if the lower incisors are placed too far labially, the tongue will be overextended anteriorly, causing the groove in the tongue to deepen, and the “s” will whistle.

It’s possible that the letter “s” will become a slurred “sh”. This occurs when the tongue is not held firmly enough in the bicuspid area, allowing air to escape at its lateral boundaries. Stigmatismus lateralis can be treated by emulating the natural prominences of the alveolar ridges in the molar areas on the denture base, providing palatal eminences on both sides.

**Figure 3:**
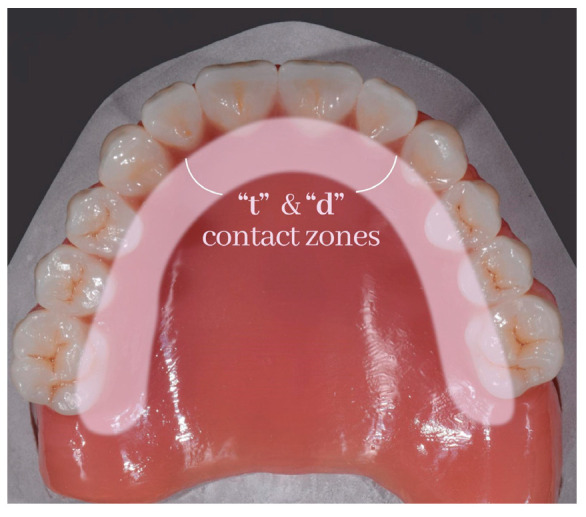
Areas involved in sounds ”t” and ”d” production.

**Figure 4:**
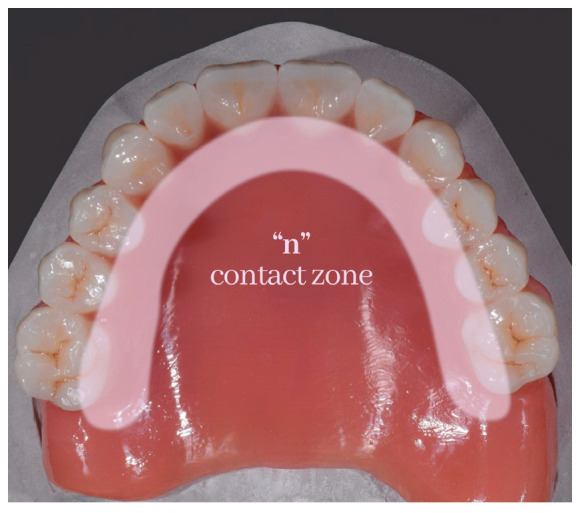
Areas involved in sound ”n” production.

Dentists are interested in the palatolingual consonant sounds of “t”, “d”, “n”, and “l” (Figure 3 and Figure 4).

The rugae region of the palate is essential to the creation of these sounds. To make such sounds, the tongue must be pressed forcefully on the front of the hard palate. As a result, the question of whether or not rugae should be replicated in the denture base emerges. According to research, the lack of rugae on the denture makes it difficult for the tongue to provide local direction [17].

If dentures are too thick, pronouncing palatolingual sounds such as “t”, “d”, “n”, and “l” might be difficult because the tongue makes early contact with the denture base. The denture should be thin where no tissue loss has occurred, such as on the palatal surface, to minimize the amount of lost tongue space.

##### Linguodental, Made with the Tongue and Teeth

Denture wearers may have trouble pronouncing the consonant “th”, which is part of the linguodental family of sounds. The sound “th” is formed when the tongue is drawn back, and the air is allowed to escape through the resulting gap. The palatogram is useful for picturing how this noise is made. The interocclusal space is used to hold the tip of the tongue between the top and lower incisal margins of the teeth. First, the tip of the tongue is retracted into the mouth, creating a passage between the palate and the dorsum of the tongue, through which air can be inhaled.

Misalignment of the front teeth (either in the interarch space or the labiolingual position) leads to acoustic deviations of the “th” sound. When pronouncing “th”, an insufficient interocclusal spacing might lead to a “biting” feeling in the tongue.

##### Labiodental, Made with the Lips and Teeth

By bringing the lower lip up to the incisal edges of the maxillary anterior teeth and forcing air through the interproximal spaces between these teeth and the irregular gaps between the edges of the teeth and the occluding surface of the lower lip, the labiodental sounds “f” and “v” are produced.

When the maxillary front teeth are positioned too far labially, the lower lip slides up beneath the incisors, causing a distortion of “f”. When these teeth are positioned aesthetically, difficulties such as these are uncommon.

##### Bilabial, Made with the Lips and Cheeks

The airflow from the lungs to the lips that produces the bilabial sounds “b”, “p”, and “m” encounters no obstacles along the way. The letters “b” and “p” are formed by closing the lips and then quickly opening them with the help of an air puff. The sound “m” is created in a similar fashion, only with some of the air being expelled nasally.

In order to produce bilabial sounds normally, the interarch distance must be right and the anterior teeth must be placed in the proper labiolingual position. If the patient’s interarch distance is too great, they will not be able to shut their lips comfortably to create an air seal. If it is too little, their lips will touch too soon. The bilabial sounds will be distorted if one of these two things goes wrong.

#### 2.2.2. Teeth

Maxillary anterior teeth in full dentures have traditionally been placed guided by linear measurements from the incisive papilla to the maxillary central incisors recorded from dentulous subjects (Table 2).

Formulating the precise location of the top anterior teeth is a crucial step in the process of designing dentures, whether full or partial. For the “5”, “55”, “f”, and “v” sounds, the “f” position [26] is achieved when the incisal edges of the maxillary central incisors contact the vermillion border of the lower lip at the confluence of the wet and dry mucosa. If teeth are too short, “v” will sound like “f” and if teeth are too long, “f” will sound as “v” [2].

#### 2.2.3. Palatal Contour

Examining the impact of different palatal contours on edentulous patients is important. Modifying the palatal vaults of maxillary full dentures has been acknowledged by the majority of authors as a means to better communication. Based on their observations of dentulous palates, some of them have proposed arbitrary adjustments to the form and thickness of the vault region [27]. Using palatograms, some have pinpointed regions of the palatal vault that, in their opinion, need adjusting.

The palatal rugae are asymmetrical and uneven elevations in the palatal mucosa behind the incisive papilla, and they can be found on both sides of the median palatal raphe in the anterior portion of the palate region. The palatal rugae provide the tongue with tactile feedback because of the abundance of mechanoreceptors inside the mucosa. As a result, they are thought to be crucial to a variety of oral processes, including speech [28].

#### 2.2.4. Denture Base

When a patient has severely diminished residual ridges, complete denture treatment is used to replace the teeth and the structures they support. It might take up a lot of space throughout this process and compromise phonetics [29]. The base of a removable denture serves several purposes, including retention, support, and stability; nevertheless, it also has the potential to significantly alter one’s speech.

Schierano et al. [30] made four different heights of palates for two patients who were able to speak normally. Subjects were recorded both with and without the artificial palates to compare the differences in their voices. A correlation between palate thickness and the degree of speech impairment was found in the study. The most noticeable change occurred while pronouncing consonants.

In order to produce the lingopalatal (anterior) sounds “c”, “d”, “t”, “n”, “s”, “z”, and “r”, the tip of the tongue makes contact with the lingual side of the anterior teeth or the most anterior part of the hard palate. The “t” in “tend” becomes unintelligible, and the “d” becomes audible if the denture base in this region is excessively thick [31].

Thicker dentures reduce air volume in the oral cavity and compress the tongue, both of which contribute to poor speech articulation. Since making palatolingual sounds requires the tongue to make contact with the palate and the alveolar process of teeth, the thickness of the denture base covering the middle of the palate is the most important factor [32,33].

When the margins are too short, the construction’s usefulness suffers, whereas when they are too long, bilabial sounds (“p”, “b”, “m”) are warped because the lips do not occlude adequately.

The distal border of a removable denture is crucial; it should cover the fovea pala-tine and sit on the “A” line for optimal function. The “a” sound phonetic test is suggested. Dentures with a properly positioned distal margin have a greater functional value and produce fewer phonetic abnormalities than those without. In the meantime, the dorsum of the tongue can get irritated by a denture that has a thick border in the posterior palatal seal area [34] or a posterior edge that is completely square rather than chamfered [30].

The palatal surface of the full denture base is normally polished to a smooth texture, unlike the uneven mucosal elevations of the palatal rugae, which may contribute to a frictionless sensation and difficulties in adjusting to diverse oral functions [35]. Consequentially, various approaches have been proposed, such as avoiding complete palatal coverage in the rugae area [36], employing a palatogramme to tailor functional palatal contours, roughening the anterior palatal surface with wrinkled wax during denture fabrication, subjecting the polished surface to airborne particle abrading, and adding palatal rugae elevations to the denture surface [18]. There are major debates about the viability of reproducing rugae on a removable denture basis [37]. Denture bases will have to be made thicker, yet tongue orientation aspects are essential for producing sounds such as the palatolingual “t”, “d”, “n”, and “l”. For proper phoneme production and speech intelligibility, it is essential that dental prostheses be as thin as feasible. Maxillary denture bases should be between 1.4 and 2 mm thick for optimal speech function [38].

Since the tongue articulates with the anterior section of the hard palate in the production of 90% of consonants, this area should not be covered by the partial denture base when possible [16]. It is crucial to maintain the hard palate’s original shape and prevent reducing the oral cavity’s volume when creating the removable denture base [39]. Sadly, there is not enough data to say for sure which method is best for patients.

The lower dental prostheses must be manufactured with concave surfaces on the tongue and cheek sides. Dentures might become uncomfortable due to pressure from the tongue on one side and the cheek mucosa on the other [40]. As a result, the dentures will be more secure, the functional suction of dental prostheses will be enhanced, and phonetic pronunciation will be enhanced since less air will be able to seep beneath the prostheses.

#### 2.2.5. Vertical Dimension, Centric Relation and Occlusal Plane

An essential and important part of making dentures is capturing the patient’s vertical dimensions and centric relationship using a variety of methods [41]. The patient’s occlusion type, vertical dimension, central occlusion, and incisal guidance may all be recorded with pinpoint accuracy using the approach proposed by Pound [42]. Calculating the “s” location is the focus of this method. When it comes to articulator structures, the “s” sound may be the most challenging to generate and the most misarticulated consonant. It has been proven that an acoustically perfect “s” sound is formed in subjects with ideal occlusion when the incisors are edge to edge, the molars are slightly separated, the mandible is lightly protruded, the tongue is consistently related to the palate and alveolar process, and the horizontal tip lies posterior to the lower incisors.

A proper “s” sound and other anterior region sounds become more challenging to produce in circumstances with excessive overjet [43,44]. It has been observed that individuals whose “s” sounds are disrupted tend to have a smaller palate than those whose “s” sounds are normal [45].

The lips must make physical contact in order for consonants such as “p”, “b”, and “m” to be formed. The lower lip contacts the maxillary incisors to alter these bilabial sounds in those with Class II malocclusion [46].

Pronunciation of the labiodental and linguo-alveolar consonants “f” and “v” is typically affected by class III malocclusion. To make these noises, one needs to bring the lower lip close to the maxillary incisors. Class III patients may display two distinct mistake patterns: the first is represented by people who make these sounds bilabially, by raising their lower lip to their upper lip and narrowing their airflow at the point of articulation; in the second, the labiodental position is inverted, with the upper lip touching the mandibular incisors [47,48].

The maxillomandibular relationship can be recorded with the help of speech in a variety of different ways. The closest speaking space of Silverman is a measurement that determines the vertical relationship of the mandible using the phonetic approach [49]. It is generally accepted that an individual’s closest speaking space remains unchanged throughout their whole life. In addition, it was mentioned that the extraction of teeth does not change speech positions [49].

It is imperative that the free-way space utilized in the centric relation technique not be confused with the closest speaking space utilized in this approach for the purpose of measuring the vertical dimension [50]. When all the relevant muscles are completely relaxed and the mandible is in its natural resting posture, the free-way space establishes a vertical dimension. When the mandible and the other muscles involved in speech are actively working to perform their full functions, the vertical dimension of the closest speaking space may be measured.

The correct alignment of the maxilla and mandible can only be accomplished by a combination of techniques, including phonetics and speech. Pronunciation tests are performed at the try-in visit after the teeth have been positioned and the wax has been molded to resemble the final denture. The dentist will realign the teeth if the patient is pronouncing certain words or numerals incorrectly [51,52].

Researchers found that difficulties pronouncing the sounds “b”, “m”, “p”, “f”, and “v” were most commonly related to an increased vertical dimension [3]. The “f” and “v” sounds of the labiodental group are useful for finding the ideal occlusal plane. To make one of these, the lower lip should meet the incisal edge of the upper front teeth.

The “v” sound changes to an “f” if the maxillary anterior teeth are located above the occlusal plane. If they are placed below the occlusal plane, then “f” will sound more like a “v” [3].

The sound “m” is the key to measuring the true height of the lower floor in a centric position. If the lips seem tight or do not make contact at all, it might be because the occlusal rims have come into contact too soon.

Pronunciation of the sounds “g”, “s”, and “z” brings the teeth close together without really touching. The dentures will actually make contact when these consonants are created if the vertical dimension of occlusion is too large, resulting in a clicking sound.

### 2.3. Speech Evaluation before and after Treatment

There are a variety of tools available for evaluating speech these days. In the event of the tooth and supporting structure loss, as well as following prosthetic rehabilitation with removable dentures, there are some that are better suited for analyzing speech alterations [12]. A standardized method for determining speech quality changes after tooth loss and further prosthodontics has not yet been established. Speech alterations before and after denture rehabilitation are a well-documented area of use for semi-standardized tools for the diagnosis of speech problems [53].

Speech intelligibility and speech quality are two aspects of speech perception that are discussed in the literature in relation to assessment following prosthodontic therapy [54].

Rodrigues et al. [54] published a study comparing how different forms of dentures affect the speech of older patients. Five speech-language pathologists analyzed videotaped speech samples from 36 patients. It was discovered that the linguodental and alveolar phonemes of those who use removable dentures were altered. There appears to be no interference with speech output due to the kind of prosthesis or its stability.

Three speech pathologists examined the speech recordings of 15 patients before and after removable denture insertion and after a week of adaptation, and Ozbek et al. [55] assessed the patients’ ability to articulate Turkish phonemes following denture application. The results indicated that some phonemes experienced articulation difficulties when a detachable partial denture was placed, while others showed great improvement.

The effects of removable dentures on the correct pronunciation of sounds in the Croatian dental and postalveolar groups were studied by Stojevci et al. [56]. Prostheses did not fully restore the articulation of postalveolar sounds, and participants who wore partial removable dentures exhibited 50% fewer distortion factors. The intensity of the formant peaks and the width of the formant bandwidths were both reduced in the groups with and without removable dentures as compared to the control group. Additionally, there was a notable enhancement in the pronunciation of the tested sounds, despite a decline in the accuracy of the articulation motions.

Articulation difficulties in denture wearers have social ramifications unrelated to their capacity to talk and communicate but rather to the discomfort that discourages them from engaging with others. Patients with articulation disorders are more likely to experience mental health issues [57]. Professional speakers have an even more difficult time with this since even small mistakes in their speech creation can have devastating effects on their careers [10,58].

Despite meticulous attention paid to the recording of maxillomandibular jaw relations, the positioning of the occlusal plane, and the arrangement of the anterior and posterior teeth, some patients experience speech difficulties after receiving their final dentures [59,60]. This is because the tongue and lips interact differently with wax (used during the trial stage) compared to the final dentures, and this has an effect on how speech difficulties are evaluated. The excessive saliva production that follows the placement of new dentures is another factor. The ability of the patient to adapt is usually sufficient to achieve satisfactory speech.

The works of certain writers highlight the importance of phonetics for patient satisfaction. The third primary goal for making a denture is the correction of speech defects due to the partial or complete loss of natural teeth in patients in compliance with phonetic requirements [61].

### 2.4. Speech Quality and Intelligibility

Because every language has its own unique phonetic system, it is imperative that the evaluation of a patient’s speech quality and intelligibility following the loss of teeth and their subsequent replacement be carried out in the patient’s native language [62]. Intelligibility and quality of speech are the two aspects of speech perception that have been researched and written about in the literature [63,64].

Speech pathology evaluations are among the most common and widely used assessments of both the quality and intelligibility of spoken language. For more trustworthy outcomes, many specialists’ evaluations are necessary. Direct speech samples, as well as audio or video records of the speech, produced in line with standardized speech pathologist examinations, may be utilized in order to carry out speech analysis [7,65].

On the other hand, this approach does have a few shortcomings, one of which is that its objectivity is dependent on the experience, acute hearing, and maybe psychological perception of the expert [66]. Essentially, it is a style of research that relies on the subjectivity of experts.

In most cases, the loss of teeth, together with its subsequent effects and future prosthetic rehabilitation, does not have an impact on vowel production [12,66]. On the other hand, consonants are often affected [67,68].

It is very easy for degenerative diseases to affect the speech mechanism, and the loss of teeth and supporting structures, in addition to affecting masticatory efficiency, alters the speech articulatory mechanism. This can cause speech disorder by affecting the speech pattern, significantly lowering the intelligibility of speech in such patients through its impact on articulation [69].

Because removable dentures reduce the amount of space in the oral cavity, a number of researchers believe that dentures have a detrimental impact on a person’s ability to produce clear speech. An investigation of clinical cases reveals that the loss of teeth and prosthetic rehabilitation with removable dentures are more likely to have an effect on sibilants (S, Z). The term “sigmatism” refers to this form of altered pronunciation in the field of speech pathology [70].

It has traditionally been thought that speech will ultimately follow the simple replacement of teeth and that it is the patient’s responsibility to fine-tune speech with practice over the course of time, or that it is entirely acceptable to have some form of disparity in speech intelligibility. However, recent research has shown that this assumption may not be accurate [31]. Table 3 below summarizes some of the most important studies conducted during this time that dealt with articulation disorders and acoustic alterations.

## 3. Discussion

Even more so than intelligence, the ability to communicate with one another is what sets the human species apart from all others. It is a highly developed pattern of coordinated neuromuscular activity that has been learned over time and is carried out automatically and subconsciously. Loss of teeth and supporting structures, in addition to affecting masticatory efficiency, alters the speech articulatory mechanism, can cause speech disorder by altering the speech pattern, and significantly reduces the intelligibility of speech in such patients due to its impact on articulation [75].

When the mechanical, aesthetic, and phonetic components of a denture work together in unison, we may say that the denture is successful. Patients often adjust well to utilizing full dentures in their speech, and any initial challenges should be manageable and not cause long-term damage. Even though there have been advancements in the manufacturing of full dentures and procedures, the number of patients who use removable dentures has increased. The biofunctional prosthetic system, which focuses on bilateral balanced articulation and is the most sophisticated approach utilized today [76], is the method that is currently used. Because of its capacity to manufacture dentures that really resemble the natural parts they substitute while also satisfying the cosmetic, functional, and phonetic needs of the patient, the biofunctional prosthetic system (BPS) is also known as the “biogenic” or “biofunctional” prosthetic system [77]. This is due to the BPS’s ability to meet all of these requirements simultaneously. Instead of expecting the patient to modify their skills to accommodate the denture, the denture should be crafted to accommodate the patient’s neuromuscular condition [78].

When making a complete denture, it is crucial to consider the impact of phonetics. The mental and physical well-being of the patient is tied to how successfully the healthcare provider communicates with him. The role of phonetics in denture creation has been the subject of several studies over the last few decades, and the results consistently show that speech is very helpful in creating a functional denture for the patient [4].

The placement of prosthetic teeth and the vertical dimension of occlusion have dominated the prosthodontic literature on phonetics [79]. Changes in speech have been linked to the palatal contour of full dentures, although only in a small number of scientific investigations [27,80]. Furthermore, there are contradictory studies on how denture users might enhance their communication skills.

The majority of the research done on how full denture wearers’ speech sounds has been conducted through case reports [81]. Even though the phonetic system has been linked to improvements in the occlusal vertical dimension, current clinical research on the topic has been restricted by a lack of control groups, blind evaluation, and detailed standards for evaluating the impact on masticatory function [12,82].

## 4. Conclusions and Perspectives

Speech is a complex, independent, and unconsciously performed process. When a person loses teeth and their supporting tissues, it has a significant impact on their speech pattern because of the shift in their major articulatory cavity. While other important factors such as aesthetics and function are given more weight during dental rehabilitation, phonetic assessment is often overlooked. The dentist’s clinical understanding of the phonetic element in denture fabrication should be supplemented by research in the speech science sector.

The primary foundations that determine whether a prosthodontic treatment is successful are the patient’s satisfaction with the treatment’s phonetics, aesthetics, function, and comfort. Oral rehabilitation for patients includes not only improving their appearance and their ability to masticate food but also their speech quality. The rehabilitation of the maxillary anterior teeth, in particular, plays a vital role in optimal speech intelligibility and articulation; their improvement results in a considerable increase in articulation capacity.

There is no denying the significance of phonetics in prosthodontics. Clinicians must have an understanding of speech production and how prostheses might alter natural speech.

When it comes to prosthetic dentistry, conducting an analysis of speech output requires a comprehensive knowledge of the characteristics of speech sounds. It is possible to identify the mechanisms and processes that may contribute to speech issues, and as a result, it is also feasible to make the required modifications, which may aid in achieving the speech change that is sought.

Understanding the nature of speech sounds, how they are created in a person, and the anatomic and physiologic components involved is crucial for an accurate analysis of speech production in prosthetic dentistry. Prosthodontists rely heavily on speech analysis to determine ideal tooth locations and define vertical measurements, which streamlines the denture-making process.

A person’s voice, as with their looks, may be a window into who they truly are. The individual’s mental health and social interactions may be negatively impacted by any aberration or deficiency in such characteristics. Therefore, a prosthodontist plays a crucial role in understanding the fundamental mechanisms involved in various speech disorders and providing judicious therapy for them to help an individual develop.

All these data found in the current dental literature has made it possible to come to some final conclusions:

- Alterations in the oral cavity caused by tooth loss and resorption of the alveolar ridge can produce changes in speech quality and intelligibility;
- Rationally planned and designed removable denture, made according to phonetic needs, improves patient’s speech function;
- Removable dentures’ functional value is an important factor for the restoration of the lost function, including speech.

## Figures and Tables

**Table 1:** Types of speech sounds and their mechanism of production.

Types of Speech Sounds	Anatomical Structures Involved	Sounds
Palatolingual	The tongue and hard or soft palate	S, T, D, N, L,
Linguodental	The tongue and teeth	Th
Labiodental	The lips and teeth	F, V
Bilabial	The lips and cheeks	B, P, M

**Table 2:** Studies on linear measurements from the incisive papilla to maxillary central incisors.

Study	Linear Measurements	Mean Distance ± SD (mm)
Ortman et al., 1979 [18]	The most anterior point of the maxillary central incisors to the most posterior point of the incisive papilla	12.454 ± 3.867
Lau et al., 1993 [19]	The most labial contour of the central incisors with reference to the standardized occlusal plane to the center of the incisive papilla	9.17 ± 1.11 mm
	The most labial contour of the central incisors with reference to the standardized occlusal plane to the posterior point of the incisive papilla	12.71 ± 1.49 mm
Park et al., 2007 [20]	The most labial contour of the central incisors to the posterior border of the incisive papilla	11.96 ± 1.37
Solomon et al., 2012 [21]	The incisive papilla and labial convexity of the central incisor	11.92 ± 2.1
Isa et al., 2012 [22]	The labial surface of the central incisors to the center of the incisive papilla	9.59 ± 1.00
Shin et al., 2016 [23]	The labial surface of the central incisors to the posterior border of the incisive papilla (CPIP)	11.62 ± 1.21
	The labial surface of the central incisors to the anterior border of the incisive papilla (CAIP)	5.72 ± 0.86
	The labial surface of the central incisors to the center of the incisive papilla (CCIP)	8.67 ± 0.90
Shrestha et al., 2016 [24]	The most labial contour of the central incisors to the posterior point of incisive papilla	11.59 ± 1.3
Kar et al., 2019 [25]	The anterior-most labial contour of central incisors to the midpoint of the incisive papilla	8.982 ± 0.996
	The anterior-most labial contour of central incisors to the posterior-most point of the incisive papilla	12.433 ± 1.008

SD—Standard deviation.

**Table 3:** Main articulation disorders in oral rehabilitated patients.

Study	Outcomes
Lundqvist et al., 1992 [71]	Approximately 30% of removable dentures users presented slightly distorted articulation for “s” and 5% for “t”.
Jacobs et al., 2001 [11]	“s” and “z” pronunciation difficulties in 70% of removable dentures wearers. Second most affected sound was “r”, followed by “t” and “d”.
Manders et al., 2003 [72]	Speech articulation problems in 75% of mandibular removable dentures mostly on “s”, “z”
Sansone et al., 2006 [73]	~92.86% of participants did not present phonetic alterations;
Molly et al., 2008 [57]	Distortions in articulation, mainly of “s” and “z” for removable dentures wearers.
Van Lierde et al., 2012 [10]	Most frequently phonetic disorders “s” and “t” followed by “z” and “d” in removable denture wearers and implant-supported fixed complete dentures users.
Fonteyne et al., 2019 [74]	Most frequently phonetic disorder “s” (sigmatism), followed by “z”

## Data Availability

Data sharing not applicable.
